# Comparing Implant Macrodesigns and Their Impact on Stability: A Year-Long Clinical Study

**DOI:** 10.3390/medicina60091546

**Published:** 2024-09-21

**Authors:** Julie Popovski, Mirko Mikic, Dimitar Tasevski, Sasa Dabic, Rasa Mladenovic

**Affiliations:** 1 Private Dental Practice Kozle, 1000 Skopje, North Macedonia; popovski55@gmail.com; 2 Department of Dentistry, Faculty of Medicine, University of Montenegro, 81101 Podgorica, Montenegro; mirko.mikic@t-com.me; 3 Private Dental Practice Endomak, 1000 Skopje, North Macedonia; kompjuterash@yahoo.com; 4 Private Dental Practice Implantodent, 78203 Banja Luka, Bosnia and Herzegovina; sajodab@yahoo.com; 5 Department of Dentistry, Faculty of Medical Sciences, University of Kragujevac, 34000 Kragujevac, Serbia

**Keywords:** dental implants, implant macrodesign, primary stability, secondary stability, resonance frequency analysis

## Abstract

*Background and Objectives*: The aim of this study was to clinically evaluate the primary and secondary stability of dental implants with different macrodesigns using resonance frequency analysis and to determine whether implant design and length influence implant stability. *Materials and methods*: This study included 48 healthy patients receiving dental implants, and a pre-implant planning protocol was used, which involved detailed bone analysis, clinical examinations, and Cone beam computed tomography (CBCT) analysis. The implants were of various types and dimensions (Alpha-Bio Tec (Israel), DFI, SPI, and NEO), and the surgical procedures were performed using standard methods. Implant stability was measured using resonance frequency analysis (RFA) immediately after placement and after 3, 6, and 12 months. The total number of implants placed in all patients was 96. *Results*: The average primary stability value for 10 mm SPI implants placed in the maxilla was 68.2 ± 1.7 Implant Stability Quotient (ISQ) units, while for 10 mm NEO implants, it was 74.0 ± 0.9. The average primary stability value for a 10 mm DFI implant placed in the mandible was 72.8 ± 1.2 ISQ, while for a 10 mm NEO implant placed in the mandible, it was 76.3 ± 0.8 ISQ. Based on the Friedman ANOVA test, the differences in the stability measurements for the 10 mm and 11.5 mm SPI implants and for the 10 mm and 11.5 mm NEO implants in the maxilla on day 0 and after 3, 6, and 12 months were significant at *p* < 0.05. Similarly, based on the Friedman ANOVA test, the differences in the stability measurements for the 10 mm and 11.5 mm DFI implants and for the 10 mm and 11.5 mm NEO implants in the mandible on day 0 and after 3, 6, and 12 months were significant at *p* < 0.05 (*p* = 0.00000). *Conclusions*: Universal tapered implants of the NEO type stood out as the optimal choice, as they provided statistically significantly higher primary stability in both soft and hard bone types compared to other implants. The implant length did not significantly affect this stability.

## 1. Introduction

Restoration with dental implants in patients with partial or complete edentulism is considered a highly reliable and predictable treatment option with high survival and success rates [1]. The success of implant therapy largely depends on the stability of the implant. Other factors include the jaw region where the implant is placed, the surgical technique, the type of implant, the occlusal load, and other factors related to the patient’s health status and habits [2,3].

The absence of clinical mobility of an implant after placement represents implant stability, which can be divided into primary and secondary stability. Primary stability refers to the mechanical anchorage of the implant in the bone and the absence of micro-movements, while secondary stability refers to the successful osseointegration of the implant with the surrounding bone.

Achieving primary stability is of utmost importance during implant placement. It is influenced by numerous factors, with the most significant being bone quality, implant design, and the surgical technique. In bones of lower quality, the primary stability of an implant can be increased by using an implant with a macrodesign specialized for soft bone, as well as by applying modified surgical techniques during implant placement, such as osseodensification drilling, the osteotome technique, piezosurgery, the under-drilling protocol, and the magnetodynamic preparation technique. The secondary, or biological, stability of an implant, as a result of bone regeneration and remodeling at the implant–bone interface, depends on primary stability, bone formation, and remodeling processes [4,5,6,7].

Several non-invasive clinical methods for assessing implant stability have been described, such as percussion tests, radiographic analysis, measurement of insertion torque, the Periotest, and resonance frequency analysis (RFA). The most widely used method in clinical and experimental practice is resonance frequency analysis (RFA) [8].

The resonance frequency analysis method utilizes sophisticated technology with computer-based measurements of resonance frequency that are determined by two parameters: the degree of bone density at the implant–bone interface and the level of marginal alveolar bone around the transducer. Devices using this principle consist of specific electromagnetically stimulated transducers placed in the implant. Depending on the vibration of the implant–transducer interface, the device provides a numerical value expressed as a quotient, known as the Implant Stability Quotient (ISQ), with a range oscillating between 1 and 100, where 100 corresponds to the maximum vibration [9].

As the design, shape, and dimensions of an implant can influence surgical outcomes (primary stability and bone compression) as well as biomechanical parameters (force distribution during occlusion), commercially available implant systems with various designs have been developed to provide optimal implant therapy for patients. Implant systems differ in their macrodesign (the appearance of the implant) and microdesign (the characteristics of the implant surface and the material from which it is made). The macrodesign of an implant refers to the shape and design of the threads, as well as the geometry, angle, pitch, depth, thickness (width), and spacing of the threads. The most important role of macrodesign is to ensure adequate stability after placement and to facilitate interaction with bone tissue through osseointegration [7,10,11,12].

Bearing in mind that current research in the field of oral implantology is aimed at improving the properties of dental implants at the level of microdesign and the molecular composition of the implant surface, this study began with the hypothesis that a new generation of universal dental implants with newly designed threads and surfaces with an innovative nanostructure would provide better primary and secondary stability compared to implants with simply machined surfaces that were specially designed for qualitatively different bone types.

The aim of this study was to clinically evaluate, using resonance frequency analysis, the primary and secondary stability of dental implants with different macrodesigns and to determine whether the design and length of an implant have an impact on implant stability.

## 2. Materials and Methods

This prospective experimental clinical study included 48 healthy patients receiving implants under favorable conditions. This clinical study was approved by the Ethical Committee of the Faculty of Medicine, University of Pristina, Kosovska Mitrovica (No. 04-3170).

This study’s inclusion criterium was missing teeth in the lateral region of the mandible or maxilla, with the height and width of the alveolar ridge being 1 mm greater than the diameter of the planned implant. Every patient signed a statement of consent for the procedure and completed a health questionnaire. Health conditions contraindicated for the surgical procedure, the presence of parafunctions, and poor oral hygiene were this study’s exclusion criteria.

### 2.1. Pre-Implant Preparation

A pre-implant planning protocol was conducted in accordance with the qualitative and quantitative characteristics of the bone tissue in the region of planned implantation. After obtaining a detailed medical history and conducting a clinical examination of the oral cavity, the protocol included the following:

- CBCT analysis of the bone tissue was performed.
- Before the start of implantation, every patient signed a statement (questionnaire) giving their consent for the placement of dental implants.
- The bone type was determined via digital methods using CBCT software (Ez3D-I, Vatech, Hwaseong, Korea) based on measurements of bone density in Hounsfield units (HUs).
- A virtual implant positioning therapy plan was developed using CBCT, with the criterium that more than one millimeter of bone tissue must surround the implant after placement.
- The surgical procedure was strictly conducted according to the principles of working in the bone and the prescribed implant protocol for Alpha-Bio Tec (Israel) dental implants.

### 2.2. Implants Used in This Study (Figure 1)

This study included three types of implants:

- DFI implants (specialized for hard bone types I and II);
- SPI implants (specialized for soft bone types III and IV);
- NEO universal dental implants (specialized for all types of bone).

**Figure 1:**
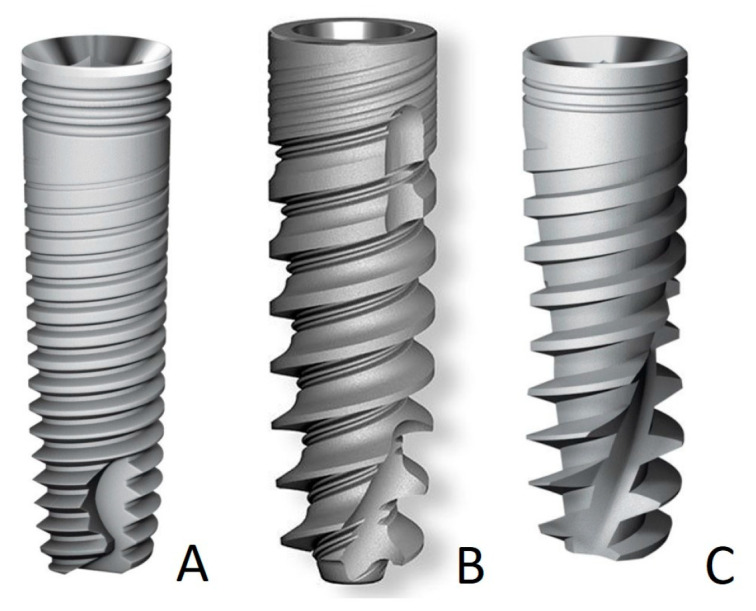
(**A**) DFI, (**B**) NEO, and (**C**) SPI implants.

The diameter of the implants was standardized to 4.2 mm, with lengths of 10 mm and 11.5 mm.

DFI-type implant: This is a classic, slightly tapered implant that is recommended by the manufacturer for hard bone types I and II. It features small and short threads in the upper body and double variable threads with cutting flutes in the apical part. It stabilizes easily, covers a large surface area, and guarantees long-term stability.

SPI-type implant: This is an original spiral tapered implant that is dynamic and powerful. It features a pronounced tapered core in the apical part with sharp and deep threads. It has high bone condensation properties that lead to high primary stability, with self-tapping and redirection capabilities. It is recommended by the manufacturer for soft bone types III and IV.

NEO universal dental implant: This is an active implant that is gentle on bone. It features a slightly tapered spiral design, a unique patented apical part with a centering function, tips for grasping, and two micro-threads. It offers optimal primary stability and high bone preservation. It is used in complex clinical cases and is recommended by the manufacturer for all types of bone.

The total number of implants placed in all patients was 96. Single implants were placed in 15 patients. In another 18 patients, two implants were placed, and in the remaining 15 patients, three implants were placed.

### 2.3. Surgical Technique for Placement

During the placement of all implants, the flap technique was used. None of the placed implants were exposed above the level of the soft tissue in the oral cavity during the healing phase (sleeping phase) until loading with a prosthetic crown. A standard surgical procedure and a standard drilling protocol were used. The insertion torque during implant placement was standardized to 35 Ncm with a torque wrench. Only successfully osseointegrated implants were considered in this study.

### 2.4. Stability Measurement

**Figure 2:**
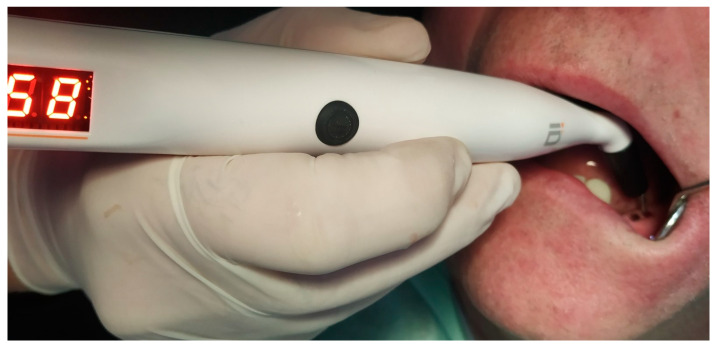
Stability measurement.

For stability testing, resonance frequency analysis (RFA) was used, and stability measurements were performed with a Penguin RFA^®^ device (Glidewell Direct, Irvine California, USA) (Figure 2). Each stability measurement was taken from the buccal and lingual sides of the upper and lower jaws immediately after placement and after 3, 6, and 12 months. A new multipeg was used for each measurement. Stability values between 55 and 85 were considered acceptable. The total number of patients included in this study was 48.

### 2.5. Statistical Data Processing

Statistical analysis was performed using the following statistical programs: Statistica 12 and SPSS 20.0. Collected data were processed using standard descriptive and analytical methods, measures of central tendency, and measures of data dispersion (means and standard deviations as well as medians and interquartile ranges). This study also used the Mann–Whitney U test, Friedman test, Kruskal–Wallis ANOVA test, and Shapiro–Wilk test. Confidence intervals (95% CIs) and statistical significance were defined for a level of error less than 0.05 (*p*).

## 3. Results

The average primary stability value for the SPI implants with a length of 10 mm placed in the maxilla was 68.2 ± 1.7 ISQ units, while for the 10 mm NEO implants, it was 74.0 ± 0.9 ISQ units.

The average primary stability value for the SPI implants with a length of 11.5 mm in the maxilla was 68.4 ± 1.2 ISQ units, while this value was 73.5 ± 1.0 ISQ units for the 11.5 mm NEO implants (Table 1).

The difference between the average primary stability values of the 10 mm SPI and NEO implants in the maxilla was significant at *p* < 0.05 (Z = −5.92815; *p* = 0.000000).

The difference between the average primary stability values of the 11.5 mm SPI and NEO implants in the maxilla was significant at *p* < 0.05 (Z = −5.92815; *p* = 0.000000). The difference between the average primary stability values of the 10 mm and 11.5 mm SPI implants in the maxilla was not statistically significant at *p* > 0.05 (*p* = 0.680052).

The difference between the average primary stability values in ISQ units for the 10 mm and 11.5 mm universal NEO implants in the maxilla was not significant at *p* > 0.05 (*p* = 0.096939).

The average primary stability value for the DFI implants with a length of 10 mm placed in the mandible was 72.8 ± 1.2 ISQ, while for the NEO implants with a length of 10 mm placed in the mandible, it was 76.3 ± 0.8 ISQ.

The average primary stability value for the DFI implants with a length of 11.5 mm in the mandible was 70.3 ± 1.1 ISQ units, while the average primary stability value for the 11.5 mm NEO implants in the mandible was 73.4 ± 1.1 ISQ units (Table 2).

The difference between the average primary stability values for the 10 mm DFI and NEO implants in the mandible was significant at *p* < 0.05 (Z = −5.84567; *p* = 0.000000).

The difference between the average primary stability values for the 11.5 mm DFI and NEO implants in the mandible was statistically significant at *p* < 0.05 (Z = −5.76319; *p* = 0.000000).

The difference between the average primary stability values in ISQ units for the 10 mm and 11.5 mm DFI implants in the lower jaw was significant at *p* < 0.05 (Z = 5.134293; *p* = 0.000000). The difference between the average primary stability values for the 10 mm and 11.5 mm universal NEO implants in the mandible was significant at *p* < 0.05 (Z = 5.608545; *p* = 0.000000).

The differences between the average stability values for the 10 mm SPI and NEO implants in the maxilla were significant at *p* < 0.05 three months (Z = −5.928115; *p* = 0.000000), six months (Z = −5.87922; *p* = 0.000000), and twelve months after placement (Z = −5.92815; *p* = 0.000000).

The differences between the average stability values for the 11.5 mm SPI and NEO implants in the maxilla were significant at *p* < 0.05 three months (Z = −5.92815; *p* = 0.000000), six months (Z = −5.86629; *p* = 0.000000), and twelve months after placement (Z = −5.92815; *p* = 0.000000).

The difference between the average stability values for the 10 mm and 11.5 mm SPI implants in the maxilla was not significant at *p* > 0.05 (*p* = 0.386477) three months after placement, but the stability was considered acceptable. The differences also were not significant at *p* > 0.05 after six months (*p* = 0.167121) and twelve months (*p* = 0.170311).

The differences between the average stability values expressed in ISQ units for the 10 mm and 11.5 mm universal NEO implants in the maxilla were significant at *p* < 0.05 three months (Z = −2.732104; *p* = 0.006293), six months (Z = −2.670245; *p* = 0.007580), and twelve months after placement (Z = 3.618749; *p* = 0.000296).

The differences in the stability measurements for the 10 mm SPI implants in the maxilla on the initial day and after 3, 6, and 12 months were significant at *p* < 0.05 (Friedman ANOVA, *p* = 0.00000) (Table 3).

The differences in the stability measurements for the 11.5 mm SPI implants in the maxilla on the initial day and after 3, 6, and 12 months were significant at *p* < 0.05 (Friedman ANOVA, *p* = 0.00000) (Table 3). The differences in the stability measurements for the 10 mm NEO implants in the maxilla on the initial day and after 3, 6, and 12 months were significant at *p* < 0.05 (Friedman ANOVA, *p* = 0.00000) (Table 3).

The differences in the stability measurements for the 11.5 mm NEO implants in the maxilla on the initial day and after 3, 6, and 12 months were significant at *p* < 0.05 (Friedman ANOVA, *p* = 0.00000) (Table 4).

The differences between the average stability values for the 10 mm DFI and NEO implants in the mandible were significant at *p* < 0.05 after three months (Z = −5.83536; *p* = 0.000000), six months (Z = −5.83536; *p* = 0.000000), and twelve months (Z = −5.92815; *p* = 0.000000). The differences between the average stability values for the 11.5 mm DFI and NEO implants in the mandible were significant at *p* < 0.05 after three months (Z = −5.92815; *p* = 0.000000), six months (Z = −5.92815; *p* = 0.000000), and twelve months (Z = −5.77350; *p* = 0.000000). The differences between the average stability values in ISQ units for the 10 mm and 11.5 mm DFI implants in the mandible were significant at *p* < 0.05 after three months (Z = 5.928150; *p* = 0.000000), six months (Z = −5.92815; *p* = 0.000000), and twelve months (Z = −5.53638; *p* = 0.000000). The differences between the average stability values for the 10 mm and 11.5 mm universal NEO implants are presented in Table 5.

Based on the Friedman ANOVA test, the differences in the stability measurements for the 10 mm and 11.5 mm DFI and NEO implants in the mandible between day 0 and 3, 6, and 12 months were significant at *p* < 0.05 (*p* = 0.00000) (Table 6).

## 4. Discussion

Several studies have documented the different stability levels of implants with morphologically different macro- and microdesigns [10,11,12,13,14,15,16]. This study included three different implants: SPI, a spiral tapered implant recommended for soft bone; DFI, a non-threaded cylindrical implant recommended for hard bone; and NEO, a universal tapered implant for all types of bone. The initial hypothesis of this study was accepted. In the presented study, a comparison of the average primary stability values of SPI and NEO implants placed in the lateral region of the maxilla (Q4 according to Norton/Gembl/0-500 HU) showed that NEO implants with nanostructured surfaces had significantly better primary stability. In research by Gomez et al. (2017), similar results were presented, showing satisfactory high values of primary stability in the maxilla even when the quality of the alveolar bone was lower [17]. Tözüm and his colleagues (2010) found that different brands of implants with threads placed in the posterior region of the maxilla achieved different ISQ stability values, with stability being proportional to the number of threads. When comparing the results in the maxilla, the average values of the SPI implants with lengths of 10 and 11.5 mm were insignificant, as were those of the NEO implants in the maxilla with lengths of 10 and 11.5 mm [18]. JJ McCullough (2017) found that macro-thread design appears to play a role in implant stability in the early post-operative healing period, as assessed via RFA [19].

Based on comparisons of the average primary stability values in the mandible (Q2/3 according to Norton/Gembl/+500-850 HU), the DFI and NEO implants placed in the lateral region of the mandible resulted in significant differences, and better primary stability was observed for the NEO implants. Our results correlate with those from a study by Lozano-Carruscal et al. (2016), who found better stability for tapered implants with sharp threads compared to cylindrical implants with rounded thread tips [20].

The comparison of the results in the mandible showed that the average primary stability values of the 10 mm DFI implants were significantly better than those of the 11.5 mm DFI implants. For the NEO implants in the mandible, the significant difference in primary stability favored the shorter 10 mm implants over the longer 11.5 mm implants. Heimes D. et al. mentioned in their research that macro-geometric characteristics (diameter and conical shape) result in a larger contact area between the bone and the implant and better primary stability, but the linear relationship between implant length and primary stability ceases to be significant at a length of 12 mm [21].

After implantation, the secondary stability of the dental implants was measured at 3, 6, and 12 months. Three months after implantation, all implants showed slight but significant increases in stability compared to their primary stability, and six months after implantation, statistically significant decreases in stability were observed compared to their primary stability, with significant increases in stability twelve months post implantation, resulting in greater stability compared to their primary stability. Our results for basic primary stability reached the highest secondary stability values in ISQ units three months after implantation in all implants. Similar results were obtained by Camaro Filho LCD et al. (2018) while examining the stability of four types of implants and the variability of secondary stability during the osseointegration process. Weekly stability values expressed in ISQ units were highest on the 91st day after implantation for all types of implants, and all implants had acceptable primary and secondary stability [22].

The overall secondary stability values of implants of the same type in the mandible depended on their lengths. When DFI implants measuring 10 and 11.5 mm were compared, the results showed a significant difference for the shorter implant. A comparison of NEO implants measuring 10 mm and 11.5 mm showed better stability for the 10 mm implants, which were 1.5 mm shorter. Argoneses et al. (2020), when analyzing the direct relationship between ISQ values and implant length, provided evidence that the highest values were shown with 10 mm implants, but significant results were only observed three months after implantation, not only in the average stability values but also in all measurements in the bucco-lingual and mesio-distal directions [23].

From the primary and secondary stability results measured using the RFA method, we can conclude that the overall average stability differences between the SPI and NEO implants in the maxilla were significant with higher stability for the NEO implants. The difference in overall average stability between the DFI and NEO implants in the mandible was significant, showing better stability for the NEO implants. Similar to our study, Monje et al. (2019) reported a strong statistical correlation between primary and secondary stability measured using the RFA method [24].

## 5. Conclusions

Based on the results of this clinical study and within its limits, as it mostly referred to different designs of the same implants, we reached the following conclusions:

- Dental implants designed for placement in all types of bone had better primary stability than implants specifically designed for bone types Q1 and Q2, where all implants were placed.
- Dental implants designed for placement in all types of bone had better primary stability than implants designed for bone types Q3 and Q4, where all implants were placed.
- Dental implants with nanostructured surfaces designed for placement in all types of bone had better secondary stability than implants designed for bone types Q1 and Q2 when all implants were placed in the lateral region of the mandible.
- Dental implants with nanostructured surfaces designed for placement in all types of bone had better secondary stability than implants designed for bone types Q3 and Q4 when all implants were placed in the lateral region of the maxilla.

## Figures and Tables

**Table 1:** The average primary stability values (expressed in ISQ units) for the implants placed in the maxilla.

Primary Stability (ISQ)						
Maxilla	Means	N	Std. Dev.	Q25	Median	Q75
SPI (10 mm)	68.2	24	1.693444	67.0	68.0	70.0
SPI (11.5 mm)	68.4 *	24	1.176460	67.0	68.0	69.5
NEO (10 mm)	74.0	24	0.907896	73.0	74.0	75.0
NEO (11.5 mm)	73.5 *	24	0.977093	73.0	74.0	74.0

* statistically significant.

**Table 2:** The average primary stability values (expressed in ISQ units) for the implants placed in the mandible.

Primary Stability (ISQ)						
Mandible	Means	N	Std. Dev.	Q25	Median	Q75
DFI (10 mm)	72.8 *	24	1.178767	72.0	73.0	74.0
DFI (11.5 mm)	70.3 *	24	1.090140	69.0	70.0	71.0
NEO (10 mm)	76.3 *	24	0.858673	76.0	76.0	77.0
NEO (11.5 mm)	73.4 *	24	1.062367	73.0	73.0	74.0

* statistically significant.

**Table 3:** The average secondary stability values (expressed in ISQ units) for the implants placed in the maxilla during three control periods: 3, 6, and 12 months.

*Secondary Stability*						
**AFTER 3 MONTHS**	**Means**	**N**	**Std. Dev.**	**Q25**	**Median**	**Q75**
SPI (10 mm)	69.5 *	24	1.560379	68.5	69.0	71.0
SPI (11.5 mm)	69.2	24	1.090140	68.0	69.0	70.0
NEO (10 mm)	74.6 *	24	0.974308	74.0	75.0	75.0
NEO (11.5 mm)	73.6 *	24	1.248187	72.5	74.0	74.0
**AFTER 6 MONTHS**	**Means**	**N**	**Std. Dev.**	**Q25**	**Median**	**Q75**
SPI (10 mm)	68.4 *	24	1.582857	68.0	68.0	69.0
SPI (11.5 mm)	67.9	24	1.361345	67.0	67.5	69.0
NEO (10 mm)	73.0 *	24	0.954585	72.5	73.0	73.5
NEO (11.5 mm)	72.2 *	24	0.977093	71.5	72.0	73.0
**AFTER 12 MONTHS**	**Means**	**N**	**Std. Dev.**	**Q25**	**Median**	**Q75**
SPI (10 mm)	69.27 *	24	1.434563	68.0	69.0	70.0
SPI (11.5 mm)	68.7	24	1.082636	68.0	69.0	69.0
NEO (10 mm)	74.37 *	24	0.806450	74.0	74.0	75.0
NEO (11.5 mm)	73.1 *	24	1.017955	72.00	73.0	74.00

* statistically significant.

**Table 4:** The Friedman ANOVA test for the differences in the stability measurements in the maxilla between the initial day and 3, 6, and 12 months.

*Maxilla*					
***SPI (10 mm)***	***Average Rank***	***Sum of Ranks***	***Mean***	***Std. Dev.***	***ANOVA Chi Sqr.***
Implantation	1.520833	36.50000	68.20833	1.693444	(N = 24, df = 3) = 48.50510 *p* = 0.00000
After 3 months	3.541667	85.00000	69.50000	1.560379	
After 6 months	1.875000	45.00000	68.37500	1.582857	
After 12 months	3.062500	73.50000	69.16667	1.434563	
***SPI (11.5 mm)***	***Average Rank***	***Sum of Ranks***	***Mean***	***Std. Dev.***	***ANOVA Chi Sqr.***
Implantation	2.291667	55.00000	68.41667	1.176460	(N = 24, df = 3) = 38.72872 *p* = 0.00000
After 3 months	3.479167	83.50000	69.16667	1.090140	
After 6 months	1.479167	35.50000	67.87500	1.361345	
After 12 months	2.750000	66.00000	68.70833	1.082636	
***NEO (10 mm)***	***Average Rank***	***Sum of Ranks***	***Mean***	***Std. Dev.***	***ANOVA Chi Sqr.***
Implantation	2.458333	59.00000	74.04167	0.907896	(N = 24, df = 3) = 49.60938 *p* = 0.00000
After 3 months	3.416667	82.00000	74.58333	0.974308	
After 6 months	1.187500	28.50000	73.04167	0.954585	
After 12 months	2.937500	70.50000	74.29167	0.806450	
***NEO (11.5 mm)***	***Average Rank***	***Sum of Ranks***	***Mean***	***Std. Dev.***	***ANOVA Chi Sqr.***
Implantation	3.104167	74.50000	73.54167	0.977093	(N = 24, df = 3) = 42.53299 *p* = 0.00000
After 3 months	3.166667	76.00000	73.58333	1.248187	
After 6 months	1.229167	29.50000	72.20833	0.977093	
After 12 months	2.500000	60.00000	73.08333	1.017955	

**Table 5:** The average secondary stability values (expressed in ISQ units) for the implants placed in the mandible during three control periods: 3, 6, and 12 months.

*Secondary Stability*						
**AFTER 3 MONTHS**	**Means**	**N**	**Std. Dev.**	**Q25**	**Median**	**Q75**
DFI (10 mm)	74.3	24	1.007220	73.5	74.0	75.0
DFI (11.5 mm)	70.7	24	0.944089	70.0	71.0	71.5
NEO (10 mm)	77.3	24	0.701964	77.0	77.0	78.0
NEO (11.5 mm)	741	24	0.880547	73.5	74.0	74.5
**AFTER 6 MONTHS**	**Means**	**N**	**Std. Dev.**	**Q25**	**Median**	**Q75**
DFI (10 mm)	71.3	24	1.434563	70.0	71.0	72.0
DFI (11.5 mm)	69.3	24	0.907896	68.5	69.5	70.0
NEO (10 mm)	75.6	24	0.710939	75.0	76.0	76.0
NEO (11.5 mm)	72.3	24	0.793999	72.0	72.0	73.0
**AFTER 12 MONTHS**	**Means**	**N**	**Std. Dev.**	**Q25**	**Median**	**Q75**
DFI (10 mm)	72.9	24	0.928611	72.0	73.0	74.0
DFI (11.5 mm)	70.5	24	0.834058	70.0	70.0	71.0
NEO (10 mm)	76.9	24	0.797414	76.0	77.0	77.5
NEO (11.5 mm)	73.1	24	0.797414	73.0	73.0	74.0

**Table 6:** Overview of the Friedman ANOVA test for the differences in the stability measurements in the mandible between day 0 and 3, 6, and 12 months.

*Mandibular*					
***DFI (10 mm)***	***Average Rank***	***Sum of Ranks***	***Mean***	***Std. Dev.***	***ANOVA Chi Sqr.***
Implantation	2.375000	57.00000	72.79167	1.178767	(N = 24, df = 3) = 63.67123 *p* = 0.00000
After 3 months	3.958333	95.00000	74.33333	1.007220	
After 6 months	1.125000	27.00000	71.33333	1.434563	
After 12 months	2.541667	61.00000	72.91667	0.928611	
***DFI (11.5 mm)***	***Average Rank***	***Sum of Ranks***	***Mean***	***Std. Dev.***	***ANOVA Chi Sqr.***
Implantation	2.645833	63.50000	70.33333	1.090140	(N = 24, df = 3) = 44.21538 *p* = 0.00000
After 3 months	3.270833	78.50000	70.75000	0.944089	
After 6 months	1.187500	28.50000	69.29167	0.907896	
After 12 months	2.895833	69.50000	70.50000	0.834058	
***NEO (10 mm)***	***Average Rank***	***Sum of Ranks***	***Mean***	***Std. Dev.***	***ANOVA Chi Sqr.***
Implantation	2.062500	49.50000	76.29167	0.858673	(N = 24, df = 3) = 54.57513 *p* = 0.00000
After 3 months	3.625000	87.00000	77.33333	0.701964	
After 6 months	1.333333	32.00000	75.62500	0.710939	
After 12 months	2.979167	71.50000	76.87500	0.797414	
***NEO (11.5 mm)***	***Average Rank***	***Sum of Ranks***	***Mean***	***Std. Dev.***	***ANOVA Chi Sqr.***
Implantation	2.791667	67.00000	73.45833	1.062367	(N = 24, df = 3) = 53.40580 *p* = 0.00000
After 3 months	3.687500	88.50000	74.08333	0.880547	
After 6 months	1.208333	29.00000	72.25000	0.793999	
After 12 months	2.312500	55.50000	73.12500	0.797414	

## Data Availability

The data are available on request from the authors.
